# Application of and Prospects for 3-Dimensional Printing in Transcatheter Mitral Valve Interventions

**DOI:** 10.31083/j.rcm2402061

**Published:** 2023-02-14

**Authors:** Yu Mao, Yang Liu, Mengen Zhai, Jian Yang

**Affiliations:** ^1^Department of Cardiovascular Surgery, Xijing Hospital, Air Force Medical University, 710032 Xi'an, Shaanxi, China

**Keywords:** mitral valve, transcatheter, interventions, 3-dimensional printing

## Abstract

Mitral valve (MV) disease is one of the most common valvular diseases that endangers health status. A variety of catheter-based interventions have been developed to treat MV disease. The special anatomical structures of the MV complex increase the difficulty of interventional surgery, and the incidence of perioperative complications remains high. With the continuous development of cardiovascular 3-dimensional (3D) printing technology and of multidisciplinary cooperation, 3D printing for transcatheter mitral valve interventions (TMVI) has become a revolutionary technology to promote innovation and improve the success rate. Patient-specific 3D printed models have been used in measuring sizes and predicting perioperative complications before TMVI. By simulating a bench test and using multi-material printing, surgeons may learn how the device interacts with the specific anatomical structures of the MV. This review summarizes relevant cutting-edge publications in this field and illustrates the application of 3D printing in TMVI with examples. In addition, we discuss the limitations and future directions of 3D printing in TMVI.

**Clinical Trial Registration:**

ClinicalTrials.gov Protocol Registration System (NCT02917980).

## 1. Introduction

Mitral valve (MV) disease is one of the most common valvular diseases to endanger health status. According to an epidemiological survey, mitral regurgitation (MR) is one of the most common heart valve diseases, and its prevalence increases with age [1]. Researchers have shown that surgical treatment of MV disease has significantly better long-term effects than treatment with drugs [2]. For many older patients who are high risk because they have multisystem diseases, a variety of minimally invasive treatments represented by transcatheter mitral valve interventions (TMVI) have always been the focus of surgeons’ explorations. However, with the continuous development of TMVI, although new devices for transcatheter mitral valve repair (TMVr) and transcatheter mitral valve replacement (TMVR) have emerged, the promotion and popularization of these techniques are still limited. This situation is related not only to the particularity of the anatomical structures of the MV but also to the difficulties involved in the preoperative selection and evaluation of patients [3, 4, 5, 6]. Considering that the MV has complex subvalvular structures and diversified lesions, transesophageal echocardiography (TEE) and computed tomography (CT) analyses have certain limitations. In addition, the development of TMVR has been slower than that of transcatheter aortic valve replacement (TAVR) in terms of interventional techniques and approved devices. The complex anatomical structures of the MV, individual differences in pathological changes, the complexity of adjacent tissues, the larger orifice area, and the higher left ventricular pressure indicate that TMVR faces more challenges related to device design than TAVR [3, 7].

**Fig. 1.**
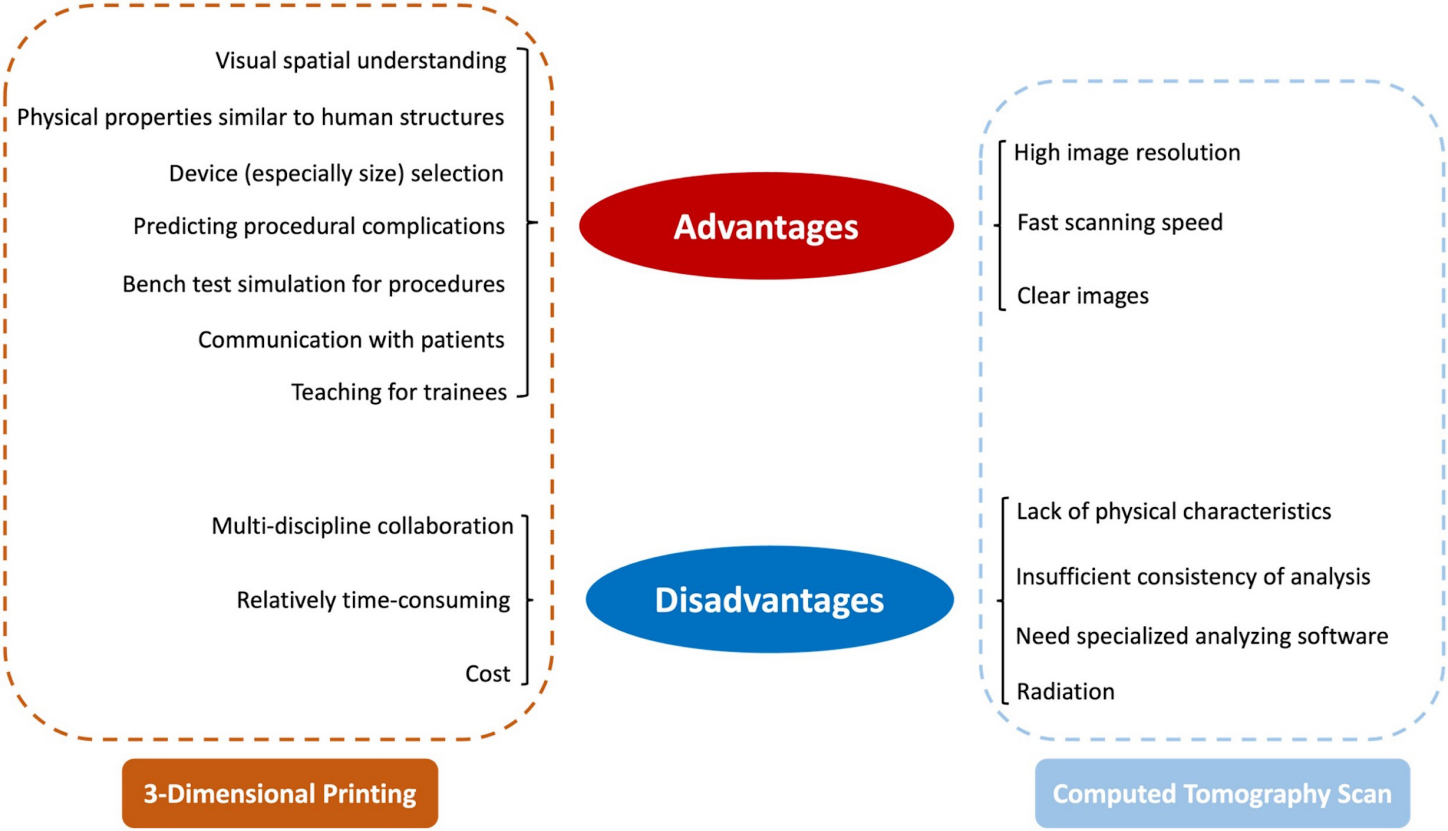
**The advantages and disadvantages between 3D printing and CT scan**.

With the continuous development of 3-dimensional (3D) printing technology and transcatheter therapy, relevant applications have become increasingly mature. The 3D printed heart models may be used to simulate the bench test, providing information that is difficult to display by traditional medical imaging (Fig. 1). In recent years, substantial progress and breakthroughs have been made in digital modeling and 3D printing of the MV, which has become an important means for evaluating TMVI [8, 9, 10, 11]. Cardiovascular 3D printing is based on traditional medical imaging, which is more intuitive and stereoscopic for complex anatomical structures and may clearly display the anatomical structures of the MV complex [12, 13]. In 2016, Little *et al*. [14] reported the first 3D printed model used for preoperative evaluation before TMVI. With the on-going progress in 3D printing, it is now possible to print different profiles in full color using a combination of different materials. Combined with clinical needs, it may display the internal heart cavity, blood vessels, valves, chordal tendineae, and other structures, providing a directive function for surgical planning [15, 16, 17, 18, 19]. This review details the role of 3D printed models of the MV in guiding and training surgeons who perform TMVr, TMVR, and paravalvular leakage (PVL) closure as an innovative technology that could significantly improve personalized patient outcomes. In addition, to accelerate the advancement of 3D printing, it is critical to understand the workflow that produces effective and functional models.

## 2. Reconstruction of 3-Dimensional Printed Model 

**Fig. 2.**
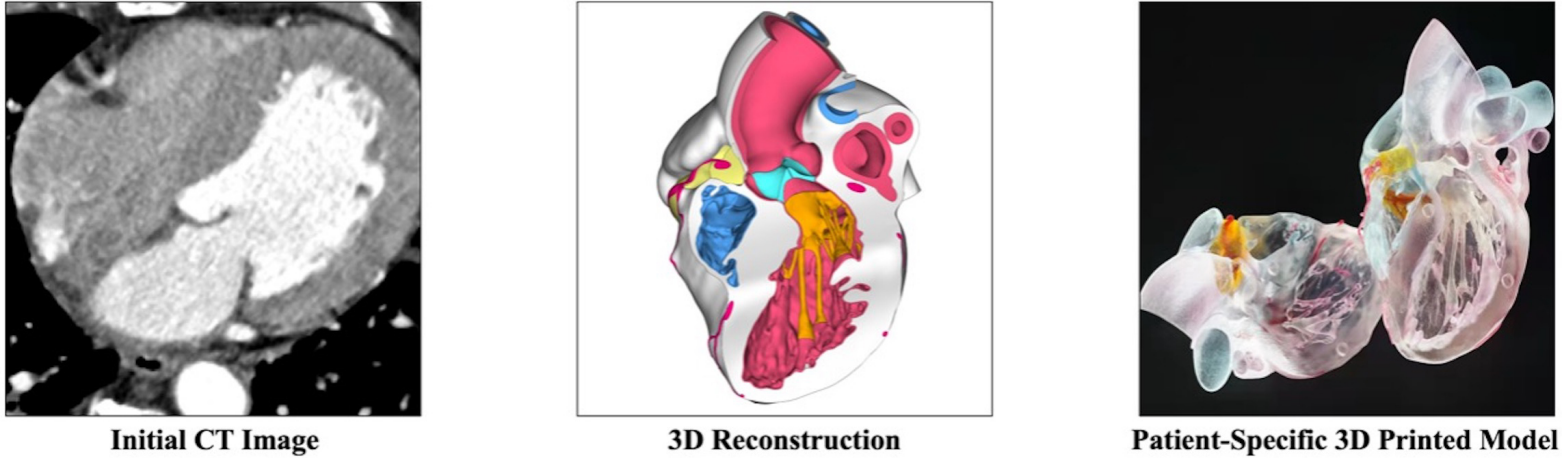
**The process of making a patient-specific multimaterial heart model**. The initial computed tomography images were collected to create a complete 3-dimensional reconstruction. Standard Tessellation Language files were imported into a 3-dimensional printer to print the model. CT, computed tomography; 3D, 3-dimensional.

Obtaining suitable imaging data is the first step of 3D reconstruction and 3D printing. First, CT data from a specific patient are imported into Materialise Mimics 21.0 version (Leuven, Belgium). The interactive function of multiplane imaging reconstruction is used to display the continuous tomography image information of three orthogonal sections (the coronal plane, the sagittal plane, and the cross plane). According to the types of MV lesions, the images of different cardiac cycles on the coronal plane of the left atrium (LA) and the left ventricle (LV) are observed to select the best image sequences. After the comparisons and confirmations are completed, the contour area is reconstructed to obtain the initial 3D model of the MV, and the collected images are converted to the standard format of the Digital Imaging and Communication of Medicine (DICOM) for storage. Secondly, the MV morphology is reconstructed comprehensively using Materialise 3-matic software (Materialise, Leuven, Belgium). Different parts of the digital model are distinguished by different colors to represent the multidimensional structural information of each part. Finally, the digital model is exported to the Standard Tessellation Language format. The Standard Tessellation Language files are imported into a Stratasys Polyjet 850 multimaterial full-color 3D printer. Depending on the type of 3D printing used, flexible materials such as resins may be used to print the models. Calcifications or stents may be printed by combining materials with different degrees of stiffness, such as polybutylene terephthalate, acrylonitrile butadiene styrene, and polyamide (Fig. 2). However, cardiovascular 3D printing is still in its infancy and needs to be further developed [20].

## 3. Application of the 3-Dimensional Printed Pulsatile Simulator

**Fig. 3.**
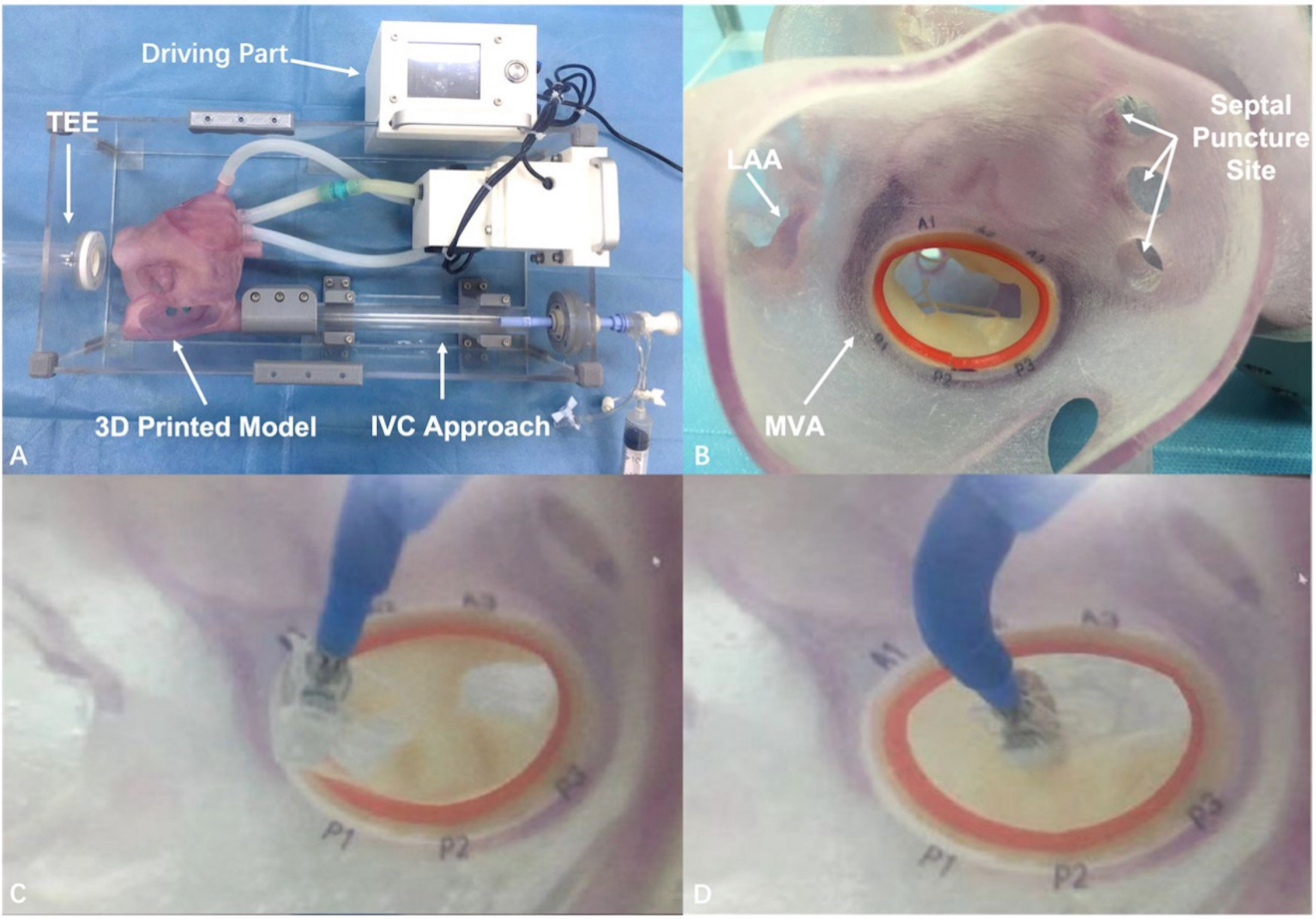
**Transcatheter edge-to-edge repair was simulated by a 3-dimensional printed pulsatile simulator**. (A) The simulator was composed of the working segment and the driving segment. (B) The anatomical structures were printed clearly so the surgeons could simulate procedures intuitively and accurately. (C) The stent was inserted into the left atrium. (D) The stent was adjusted to the ideal position of anterior 2-posterior 2. 3D, 3-dimensional; IVC, inferior vena cava; LAA, left atrial appendage; MVA, mitral valve annulus; TEE, transesophageal echocardiography.

Due to the difficulty of performing TMVI, the development of a pulsatile simulator for teaching and simulation-assisted learning is of great significance for surgeons and students. The pulsatile simulator of TMVI based on 3D printing comprises two segments: the working segment includes the inferior vena cava approach, the complete four chambers of the 3D printed heart, the TEE approach, the puncture position of the atrial septum, the MV leaflets, and the related sub-valvular structures; the driving segment includes the circulating pump, the complete connection loop, and the control system (Fig. 3). There are preset puncture openings at different locations in the atrial septum, which may simulate transcatheter edge-to-edge repair (TEER) at different puncture sites. The anterior zone and the posterior zone of the MV are color-coded with the chordae tendineae. By adjusting the driving segment and relying on the internal circulation, MV prolapse and MR may be simulated, and prolapse in different areas and the degree of prolapse may be controlled. A significant characteristic of the pulsatile simulator of TMVI based on 3D printing is that it can be clearly observed under TEE. At present, the pulsatile simulator of TMVI based on 3D printing can be used for simulations performed during the bench test by a variety of transcatheter devices. Through the simulations on the pulsatile simulator, trainees may advance their understanding of the related procedural skills, shorten the learning curve, improve their procedural abilities and the success rate of TMVI.

## 4. 3-Dimensional Printing and Transcatheter Mitral Valve Repair

Since the first TMVr was applied in clinical practice in 2003, more than 150,000 patients worldwide have had the operation [21]. However, for several pathophysiological reasons such as calcification, MV prolapse, or MV splitting, the actual anatomical structures of the MV are often very different from the typical anatomical structures, which makes the procedures challenging. At the same time, the clinical evaluation of TMVr depends mainly on the severity of the postoperative paravalvular leakage (PVL) as quantified by TEE. However, the ultrasonic artifacts caused by implanted devices and the common multipoint PVL make an accurate quantification challenging. Therefore, the simulation of MR in patients may provide the assessment for blood flow measurement.

The special anatomical structures of the MV lesions can seriously affect the process of capturing leaflets, so the 3D printed model may play an important role. Accurate 3D printed models of the MV, as a good training tool, may help surgeons and trainees interact with the realistic models before actually performing the TMVr and improve the success rate and accuracy of the procedures. The patient-specific 3D printed models are used to fully display the anatomical structures of the MV and to enable comprehensive planning of possible complications during TMVr to guide the selection of patients and ensure the appropriate implementation of the procedures [13, 22, 23, 24].

At present, a multi-material 3D printed model of the MV can be reconstructed using imaging data, and the pulsatile simulator may be constructed. Vukicevic *et al*. [12, 25] developed a multimaterial 3D printed model of the MV for simulations during the bench test and planning of TEER using the MitraClip (Abbott Vascular, Santa Clara, CA, USA). They also reported two patient-specific conditions: (1) which MR was suitable for TEER and (2) MV perforation with the percutaneous occluders due to endocarditis [20]. The delivery system was inserted via the LA; the gripper was perpendicular to the anastomosis of the anterior lobe and the posterior lobe; then the clamping arm was lowered to clip the MV leaflets, and finally the device was withdrawn from the LV to clip the leaflets [26, 27, 28]. The MitraClip may be better understood through using the 3D printed model. The surgeons may master capturing skills, and the procedural skills could be maintained during the simulation to ensure the complete clamping of leaflets [14].

**Fig. 4.**
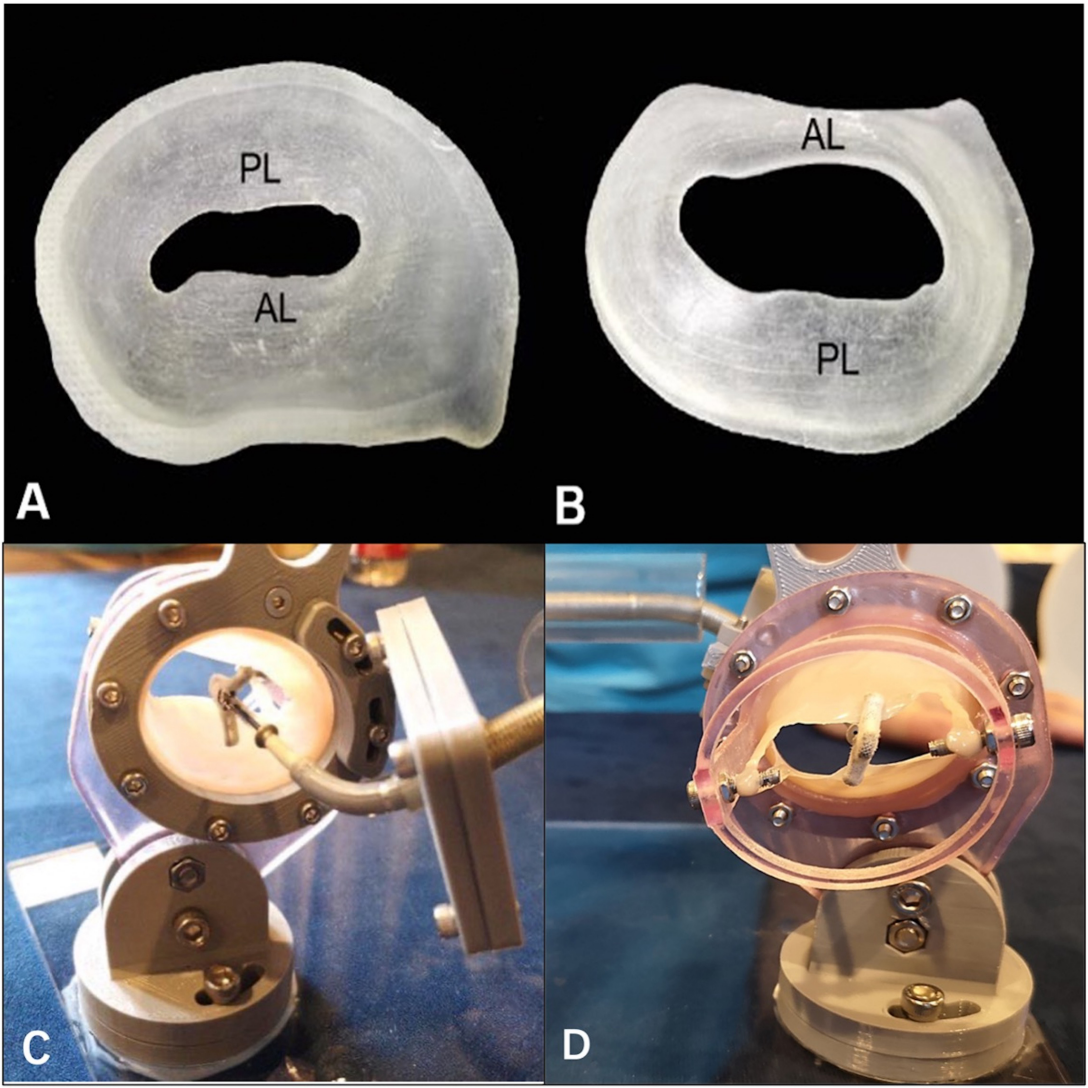
**The 3-dimensional printed mitral valve model was used to simulate transcatheter edge-to-edge repair during the bench test**. (A) The 3-dimensional printed mitral valve model from the left atrium plane. (B) The 3-dimensional printed mitral valve model from the left ventricle plane. (C) The MitraClip (Abbott Vascular, Santa Clara, CA, USA) was bent and positioned in the left atrium view. (D) The MitraClip was clamped to the leaflet in the left ventricle view. AL, anterior leaflet; PL, posterior leaflet.

Because it is built from computed tomography angiography (CTA) and TEE data, the patient-specific 3D printed model can be used to restore the true anatomical structures of the MV, which may not only be displayed but may also show the opening and closing of the leaflets under pulsation. In addition, the 3D printed model is compatible with TEE, which may be used to obtain clear images. At the same time, several cameras can be inserted, which would enable the participants to observe the whole implantation process from multiple angles, thus helping trainees to enhance their understanding of the MV, become more familiar with the procedures, evaluate the curative effect, shorten the learning curve, and reduce the number of possible complications [22, 23]. The pulsatile simulator based on the patient-specific 3D printed model further improves the function of 3D printing, which is to closely reflect the expected effect of the procedures and provide a powerful method to carry out personalized evaluations of TMVr (Fig. 4).

## 5. 3-Dimensional Printing and Transcatheter Mitral Valve Replacement

**Fig. 5.**
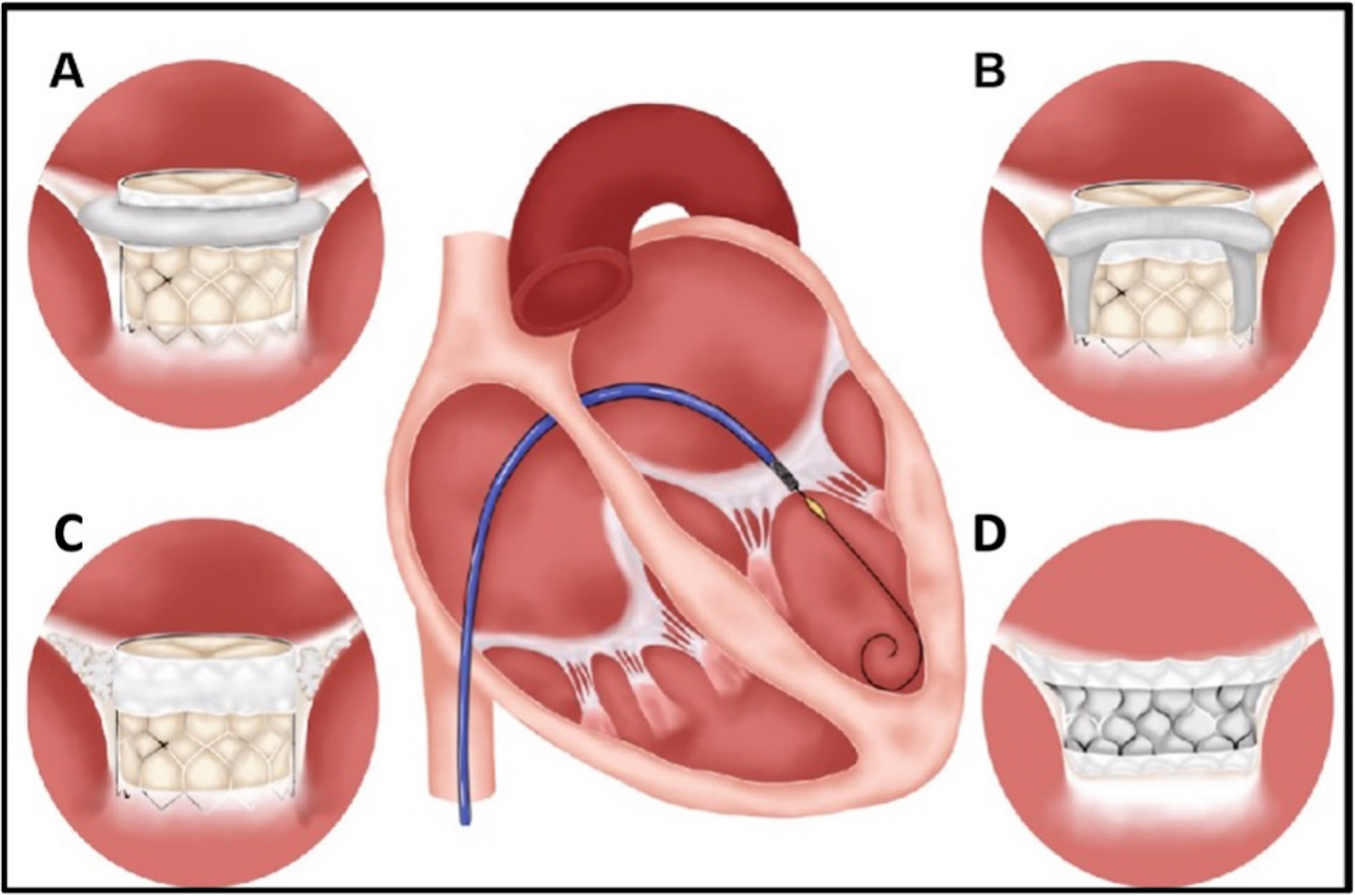
**The classification of transcatheter mitral valve replacement**. (A) The mitral valve in a ring implant. (B) The mitral valve in the valve implant. (C) The implant in a case of mitral annulus calcification. (D) Implant in a native mitral valve.

The emergence of TMVR provides a new treatment method for a large number of patients who could not undergo conventional operations [29]. Compared with TAVR, TMVR involves more problems and challenges because of the following special anatomical structures of the MV complex. Nowadays, TMVR technology may be divided into four major categories according to the MV lesions (Fig. 5). (A) Mitral valve-in-valve implant. The mitral valve-in-valve implant has been successfully used to treat degenerated valves and has emerged as a promising strategy for a failing bioprosthesis [30, 31, 32, 33, 34, 35, 36]. When making a preprocedural plan, the location of the transseptal puncture is the key that the surgeons must solve. In addition, the pressure that causes the MV to close is systolic. The excessive pressure may lead the stent to shift more easily at the annulus of the MV, so a relatively larger stent is needed to solve that problem. (B) Mitral valve-in-ring implant. It is currently suggested that a transcatheter mitral valve-in-ring implant has been considered a new alternative treatment for MV diseases [37, 38, 39]. During the procedure, the stent should be released along the central line of the MV ring [2]. In order to anchor the stent and prevent several possible perioperative complications, it is recommended that the stent be 10% larger than the inner diameter of the bioprosthesis [31]. However, the larger stent may lead to the leaflets not being able expand fully. Therefore, it is of great significance to carry out individualized preprocedural simulations, accurately evaluate the anchor position of the stent, the possible location of the PVLs, and then help surgeons improve the surgical plan and formulate risk management measures. (C) Mitral annulus calcification (MAC) implant. MAC refers to a lesion of the MV characterized by annular fibrosis and degenerative calcification. The lesion is associated with endocarditis, coronary heart disease, valvular heart disease, and congestive heart failure and may lead to mitral stenosis or MR in severe cases [40, 41]. When treating patients with MAC, TMVR often leads to complications, such as LVOT obstruction, PVL, and aortic root rupture. To understand and predict these complications will help optimize the therapeutic effect of TMVR [42, 43]. Part of the challenge is related to the complexity of the MV device and the specific anatomical structures of the LV [20]. (D) Native mitral valve implant. In Europe, the prevalence of MR was 24.4% and that of severe MR was 5.9% in people screened by echocardiography [44]. The prevalence of MR in the United States is 6.4% in people aged 65–74 years and 9.3% in people aged above 75 years [1]. Although surgical mitral valve replacement is the standard treatment for relieving MR, about 50% of patients with severe MR may not undergo surgical mitral valve replacement because of serious comorbidities. TMVR is challenging due to the particularity of the anatomical structures and the complexity of the adjacent tissues, such as peripheral conduction bundle branches and coronary artery circumrotatory branches. In addition, because of the good coaxiality with the MV, the transapical approach is the preferred approach for TMVR, so the determination of the puncture point is extremely important.

**Fig. 6.**
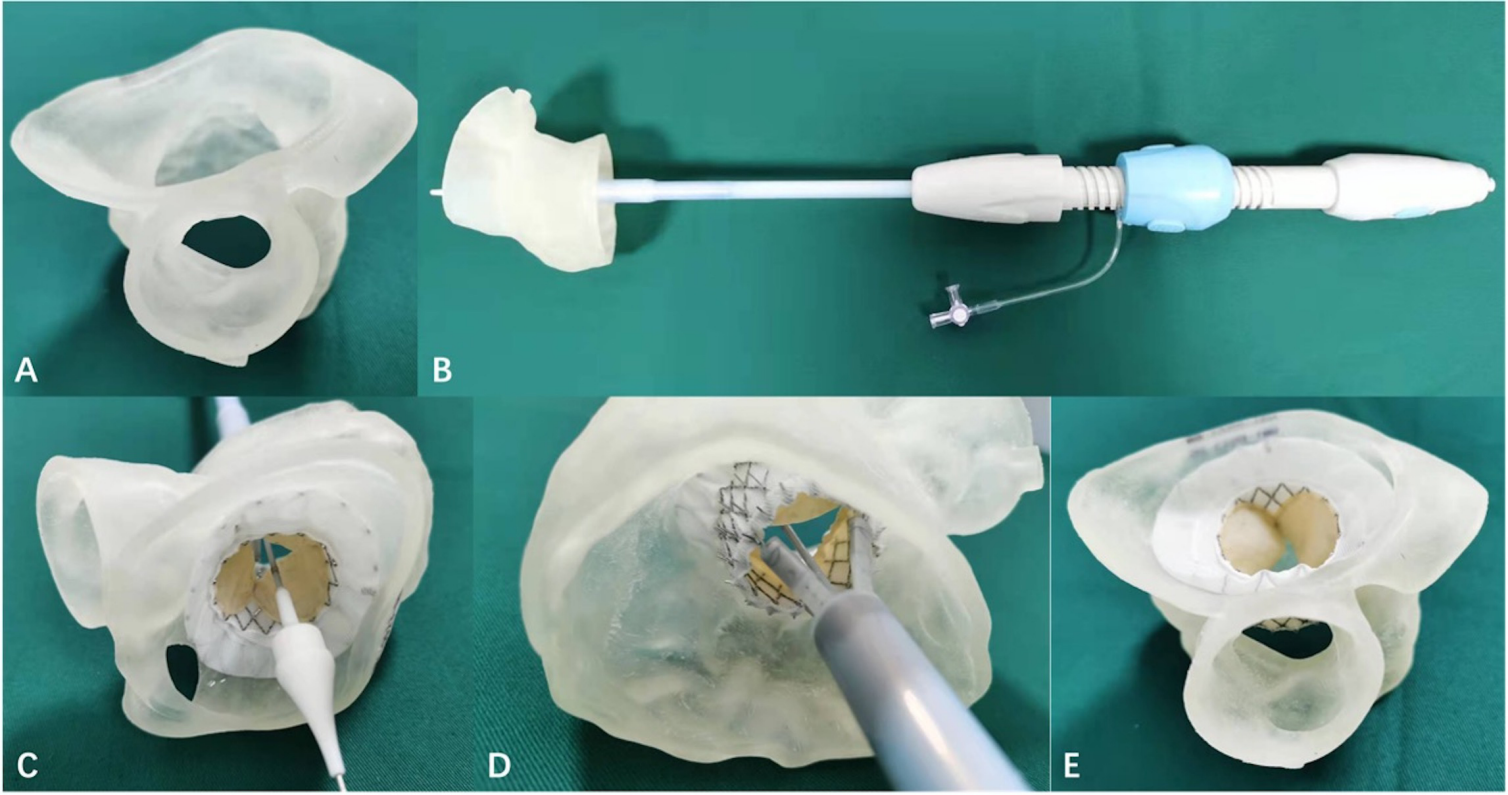
**The simulation of transcatheter mitral valve replacement during the bench test to make the procedural plan and determine the appropriate size of the stent**. (A,B) The patient-specific 3-dimensional printed mitral valve model and the delivery system used for transcatheter mitral valve replacement simulation (Microport, Shanghai, China). (C,D) The implant process in the left atrium view and the left ventricle view, respectively. (E) The 3-dimensional printed model in the left ventricle view after the simulations.

Therefore, preoperative CTA and 3D reconstruction of the anatomy of the patients, a detailed evaluation of the indications, and a preliminary simulation are particularly important for the successful implementation of various types of TMVR. Currently, TEE and CT are the main imaging methods used to plan the operation [26, 45, 46, 47, 48]. Preoperative CTA images may be used digitally to simulate the recommended projection angles of the different approaches through the transapical and transfemoral approaches [49, 50, 51, 52, 53, 54, 55]. However, the medical imaging methods above have limitations to formulate accurate surgical plan. The patient-specific 3D printed models may help surgeons select the appropriate puncturing position and avoid the related coronary arteries, the chordae tendineae, and the papillary muscle. Depending on the angle between the apical puncture, the atrial septal puncture, and the MV annulus in different patients, surgeons may pre-model and increase the coaxiality of the device. In a previous study, we evaluated the size and height of the annulus and the best projection angle of the released valve using 3D printed models [56]. At the same time, simulations performed during the bench test may be used to evaluate the feasibility of procedures, surgical strategies, technical points, prevention of complications, and postoperative evaluation, thereby resulting in the accumulation of valuable experience for the successful implementation of TMVR (Fig. 6) [57, 58]. In addition, 3D printed models may provide useful guidance for the predicted size of the neo-LVOT and help surgeons to select the appropriate stent and expected position of the stent [11, 13, 14, 20, 59, 60, 61, 62, 63]. Due to the significant advantages such as personalization and repeatability, 3D printing will certainly play a more important role in TMVR.

**Fig. 7.**
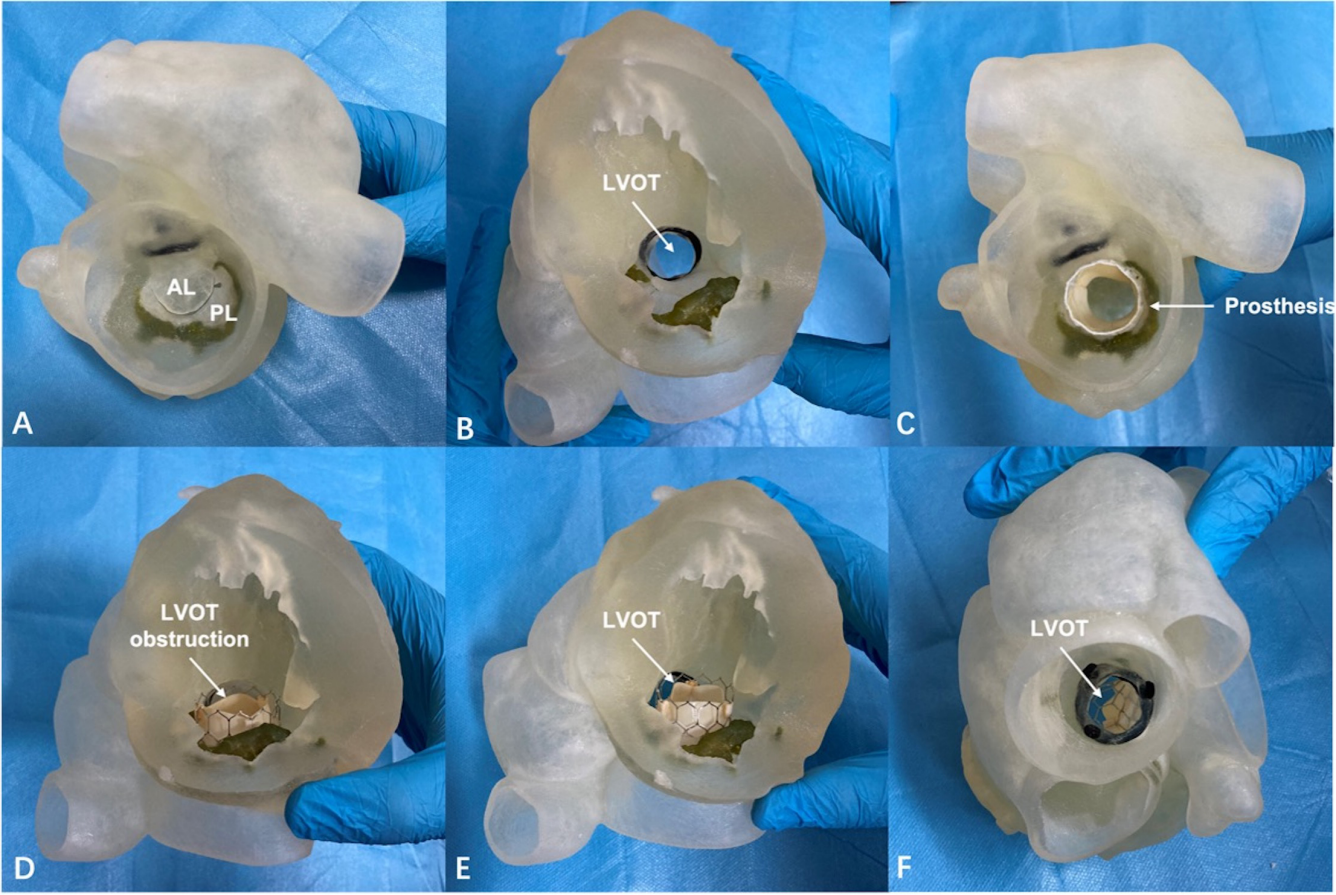
**The 3-dimensional printed model was used to assess the risk of left ventricular outflow tract obstruction after transcatheter mitral valve replacement**. (A) The distribution of the mitral annulus calcification was observed clearly from the left atrium plane. (B) The left ventricular outflow tract was observed from the left ventricular plane. (C) The bioprosthesis was implanted during the bench test. (D) Left ventricular outflow tract obstruction occurred due to anterior leaflet displacement after the simulation. (E) The patency of the left ventricular outflow tract after anterior leaflet resection. (F) The left ventricular outflow tract was observed after anterior leaflet resection from the ascending aorta plane. AL, anterior leaflet; LVOT, left ventricular outflow tract; PL, posterior leaflet.

In addition, the main intraoperative complications in TMVR could be predicted before procedures. (A) PVL. The annulus is deformed with the periodic beats and patients exhibit significant individual differences in the anatomical structures. The characteristics of the annulus render preoperative stent selection and TMVR procedures extremely challenging. To ensure that PVL does not occur after TMVR, it is necessary to select the stent that best fits the patient’s MV annulus. (B) Left ventricular outflow tract (LVOT) Obstruction. TMVR is likely to lead to LVOT obstruction, which may result in arrhythmia and congestive heart failure, especially in older patients with severe MAC [64, 65]. Viewing the 3D printed model of the patient-specific left heart and the simulations made during the bench test may reveal the characteristics of patients at risk for LVOT obstruction, which could provide an obvious advantage when determining the type of procedure and anticipating possible problem areas, thereby leading to a reduction in intraoperative complications. Therefore, individualized preoperative simulation using a personalized 3D printed model is of great significance to accurately predict the procedural results and the possible locations of the PVLs and to help surgeons improve operative strategies. Eleid *et al*. [65] first reported in 2016 that a patient-specific 3D printed model was successfully used to predict PVL and LVOT obstruction after TMVR [66, 67, 68, 69]. They found that the preoperative evaluation results based on the 3D printed models accurately reflected the locations and the size of the PVLs, and enabled the surgeon to directly evaluate the rationality of the LVOT and the selection of the stent [65]. According to the results of simulations, the selection of an appropriate bioprosthesis and a surgical plan could be determined for patients with MAC who undergo TMVR (Fig. 7).

## 6. 3-Dimensional Printing and Mitral Paravalvular Leakage

**Fig. 8.**
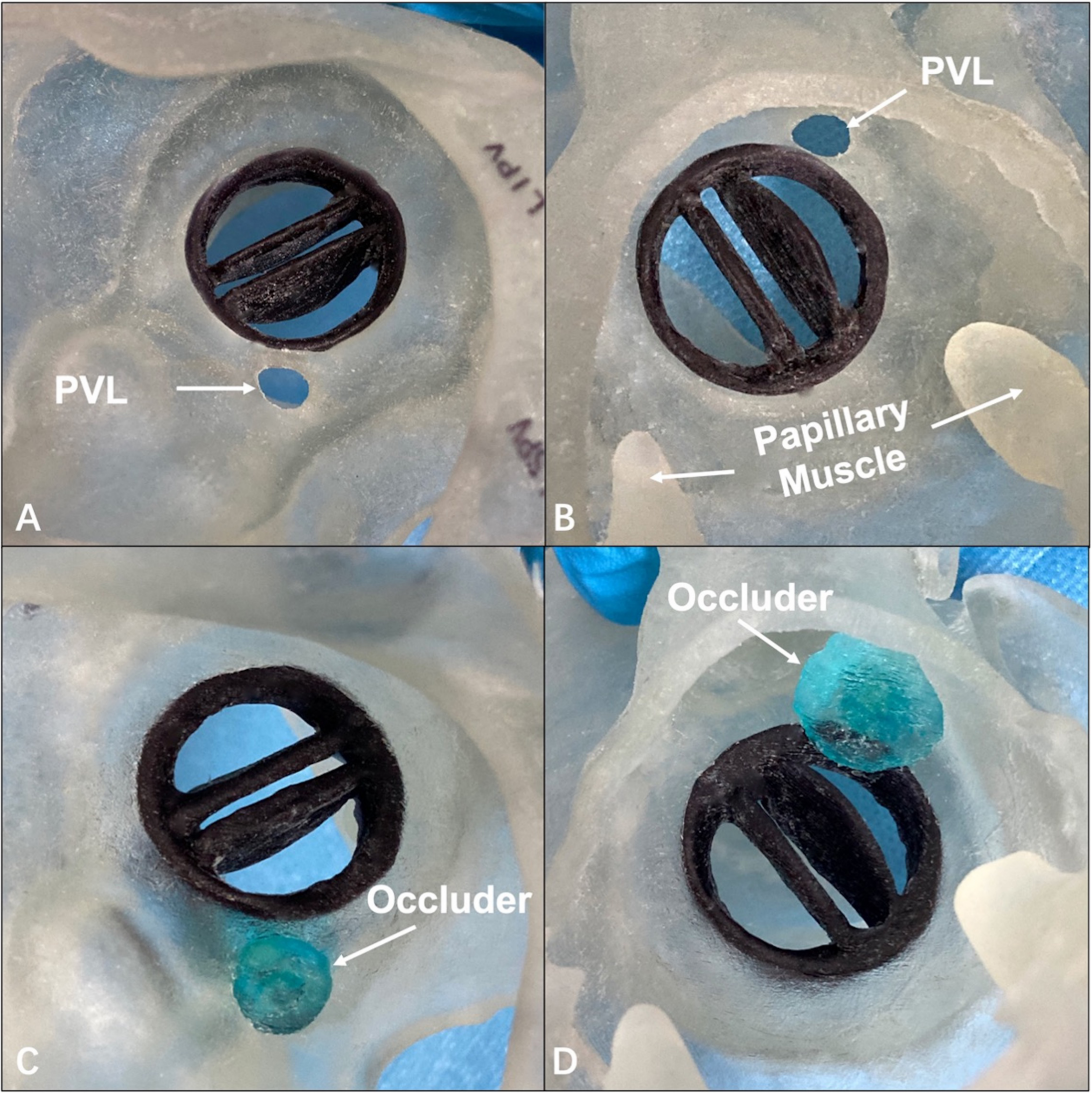
**The 3-dimensional printed mitral valve model was used to demonstrate the effect of paravalvular leakage occlusion**. (A,B) Paravalvular leakage was observed from the left atrium plane and the left ventricle plane, respectively. (C,D) The occluder was implanted during the bench test and was observed from the left atrium plane and the left ventricle plane, respectively. PVL, paravalvular leakage.

PVL is a unique complication after a heart valve replacement procedure. It is caused by a variety of events, such as annular rupture, annular tear at the suture site, and calcification of the MV annulus. Previous studies have shown that the incidence of PVL after surgical mitral valve replacement is about 7–17% [70]. Severe PVLs may cause a variety of complications, such as heart failure, arrhythmia, hemolysis, and endocarditis. As usual, the primary treatment for PVL is the operation, but the risk of a surgical reoperation is very high and the risk of death increases accordingly. In some patients, the PVL is located in a special position; in other patients, the larger heart cavity makes it difficult for the guide wire to pass through the leak. During the operation, the PVL is repaired by establishing multiple surgical approaches. The occlusion of the PVL of the MV requires a high level of surgical skill. When the leak is small and the guide wire is difficult to reach, the X-ray exposure time and the amount of radiation are increased significantly. TEE may accurately note the location of the PVL and quantitatively evaluate the severity, but the presence of artifacts often makes it is difficult to judge accurately the size of the leak [71]. Preoperative CTA evaluation may help determine the shape and size of the PVL. CTA images can, however, contain several artifacts that affect the accuracy of the PVL evaluation. Variations in the size, position, and morphology; in the approach; and in the types of occluders used are not identical. Conventional imaging modalities, such as CTA and TEE, make it difficult to fully identify the various factors and related risks after the occluders are implanted. As 3D printing matures, surgeons may reconstruct the MV and the adjacent anatomical structures and print the 3D printed model according to the patients’ CTA data, which will help them identify the position of the PVL by simulating different types of occluders and directly observing the relationship between the MV and the occluders [72]. At the same time, surgeons may also use 3D printed models of PVLs to conduct preoperative simulations during the bench test and develop personalized surgical plans, the goal being to reduce the operating time and the amount of radiation used during the procedure (Fig. 8).

## 7. Limitations

Although 3D printing is being used with increasing frequency in TMVR, some problems still need to be addressed before the technology may be used more widely: (1) The accuracy of 3D printed models needs to be improved. Current software is used to print models of the heart without considering its dynamic characteristics. Therefore, the models do not track the dynamic changes that occur after the stent is deployed, such as tissue deformation, device tilt, and insufficient expansion, which may result in measurements being underestimated or overestimated. (2) The characteristics of the anatomical structures and mechanical properties need to be improved. Although current 3D printing technology can print multiple materials with various properties, which may reflect the anatomical structures and mechanical characteristics of pathological conditions to a certain extent, it still lags behind in recreating the real properties of heart. (3) The process of printing the models needs to be improved. Professionals often need careful segmentation to ensure that the anatomical details are captured, and formulating high-quality 3D reconstructions is time-consuming.

## 8. Future Directions

TMVI is a revolutionary technological breakthrough for the treatment of MV diseases that differs from traditional surgical valvular procedures: Surgeons using this technology must develop personalized surgical strategies based on the evaluation of preoperative images and fully understand the dynamic 3D anatomical structures of the MV combined with intraoperative guidance using digital subtraction angiography. Traditional imaging techniques based on the concept of disease diagnosis cannot fully meet the needs of TMVI. When the clinical applications of TMVI were first introduced, 3D printing began to play a role in many aspects, including helping surgeons to accurately formulate surgical strategies, select appropriate stent types, and assist in the development of new devices. Moreover, as the rapid development of TMVI continues, new medical imaging requirements are continuously being requested, such as more accurate reconstructions and more refined printing [66], computational fluid dynamics and fluid-structure interaction [73, 74, 75, 76, 77], patient-specific printed bioprosthesis [78, 79, 80, 81], virtual reality [82], 3D real-time image fusion [83], and artificial intelligence [84]. In the future, the integration of 3D printing, surgical simulation, CFD, and AI will be an important developmental direction for clinical training, device research, and precision medicine in the future.

## 9. Conclusions

Although the application of 3D printing in TMVI is still in an early stage, the value of personalized 3D printed models in guiding the precise treatment of MV disease is clear. In the future, the application of patient-specific 3D printed models to bioprostheses and repair devices will enable 3D printing to play a more in-depth role in TMVI, and precise individualized treatments guided by 3D printing and imaging technology will benefit more patients.

## Abbreviations

3D, 3-dimensional; AI, artificial intelligence; AV, aortic valve; CFD, computational fluid dynamics; CT, computed tomography; CTA, computed tomography angiography; LA, left atrium; LV, left ventricle; LVOT, left ventricular outflow tract; MAC, mitral annulus calcification; MR, mitral regurgitation; MS, mitral stenosis; MV, mitral valve; PVL, paravalvular leakage; TAVR, transcatheter aortic valve replacement; TEE, transesophageal echocardiography; TEER, transcatheter edge-to-edge repair; TMVI, transcatheter mitral valve interventions; TMVr, transcatheter mitral valve repair; TMVR, transcatheter mitral valve replacement.
